# Supplementary material for: Risk factors for sacrococcygeal pilonidal sinus: a systematic review and meta-analysis supplemented by genetic causal assessment
Source: Front Surg. 2026 Jan 7;12:1718589. doi: 10.3389/fsurg.2025.1718589 (PMC12819706; doi:10.3389/fsurg.2025.1718589)
Supplement: Supplementary file 2 [file Datasheet2.zip › Supplementary Data 2/MR_pipeline_p5e-8/leaveone_plots/ukb-d-1747_5.finngen_R12_L12_PILONIDALCYST.pdf]

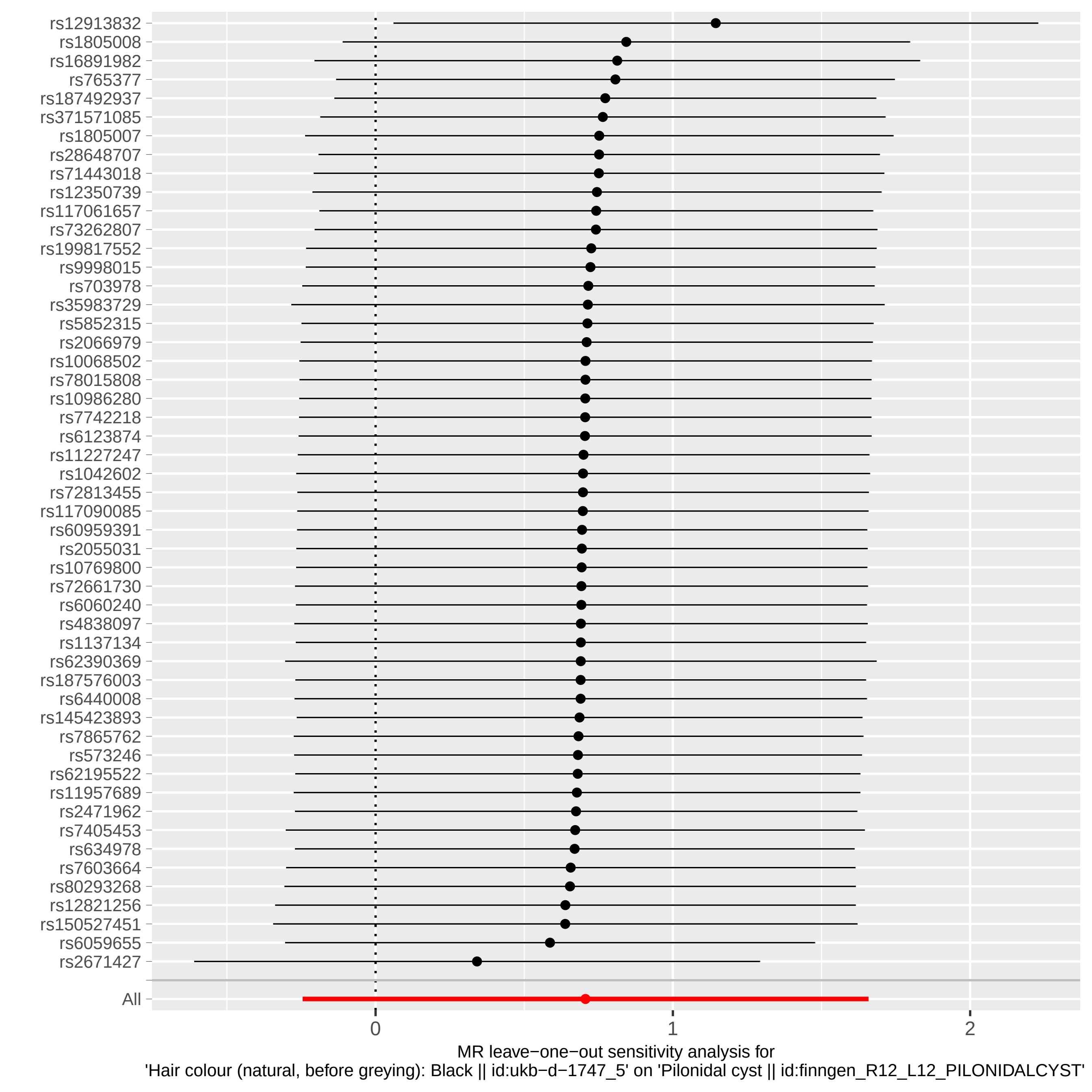
rs12913832
rs1805008
rs16891982
rs765377
rs187492937
rs371571085
rs1805007
rs28648707
rs71443018
rs12350739
rs117061657
rs73262807
rs199817552
rs9998015
rs703978
rs35983729
rs5852315
rs2066979
rs10068502
rs78015808
rs10986280
rs7742218
rs6123874
rs11227247
rs1042602
rs72813455
rs117090085
rs60959391
rs2055031
rs10769800
rs72661730
rs6060240
rs4838097
rs1137134
rs62390369
rs187576003
rs6440008
rs145423893
rs7865762
rs573246
rs62195522
rs11957689
rs2471962
rs7405453
rs634978
rs7603664
rs80293268
rs12821256
rs150527451
rs6059655
rs2671427
All
0
1
2
MR leave−one−out sensitivity analysis for
'Hair colour (natural, before greying): Black || id:ukb−d−1747_5' on 'Pilonidal cyst || id:finngen_R12_L12_PILONIDALCYST'
